# The mediating role of digital health literacy in the relationship between social support and health-promoting lifestyle among occupational populations: a cross-sectional study

**DOI:** 10.3389/fpubh.2026.1865814

**Published:** 2026-08-31

**Authors:** Yun Pan, Tantan Cheng, Tianyu Sha, Jiakui Zhao, Yeqing Gu

**Affiliations:** 1 Shanghai Huangpu District Center for Disease Control and Prevention (Shanghai Huangpu District Health Supervision Institute), Shanghai, China; 2 Key Laboratory of Health Technology Assessment of Ministry of Health, School of Public Health, Fudan University, Shanghai, China; 3 Shanghai Huangpu District Health Affairs Management Center, Shanghai, China

**Keywords:** digital health literacy, health-promoting lifestyle, mediation analysis, occupational population, social support

## Abstract

**Background:**

In the digital age, health-promoting lifestyle (HPL) among occupational populations is critical for chronic disease prevention. Social support has been recognized as a key facilitator of HPL, yet the underlying mechanisms remain unclear. Digital health literacy (DHL)—the ability to access, understand, and apply online health information—may serve as a potential mediator. However, empirical evidence on this mediation model in occupational settings is limited. This study aims to examine the mediating role of DHL in the relationship between social support and HPL among occupational populations.

**Methods:**

A multistage stratified cluster sampling method was used to recruit 1,030 occupational participants in Huangpu District, Shanghai, between July and September 2024. Data were collected using standardized questionnaires assessing social support, DHL, and HPL. Descriptive statistics, Pearson correlation analysis, and bootstrap-based mediation analyses were conducted.

**Results:**

Social support was positively correlated with DHL (*r* = 0.176, *P* < 0.001) and HPL (*r* = 0.324, *P* < 0.001), while DHL was also positively associated with HPL (*r* = 0.306, *P* < 0.001). Mediation analysis indicated that DHL partially mediated the relationship between social support and HPL (indirect effect = 0.113, 95% CI [0.065, 0.170]), accounting for 13.9% of the total effect. Parallel mediation analysis further showed that only the application dimension of DHL had a significant mediating effect (effect = 0.081, 95% CI [0.026, 0.146]), whereas the other dimensions were not significant.

**Conclusions:**

Social support is positively associated with HPL both directly and indirectly through DHL. Notably, the application of digital health information plays a key role in translating social support into health-promoting behaviors. Interventions aiming to improve occupational health should prioritize not only strengthening social support but also enhancing individuals' ability to apply digital health information in daily life.

## 1 Introduction

Occupational populations constitute a major component of the national workforce and play a crucial role in socioeconomic development. By the end of 2023, the number of employed individuals in China exceeded 740 million, accounting for more than half of the total population (1). However, with rapid industrialization and changes in work patterns, occupational populations are increasingly exposed to multiple health risks, including prolonged sedentary behavior, high work-related stress, insufficient physical activity, and irregular sleep patterns (2, 3). These factors significantly increase the risk of chronic diseases and other adverse health outcomes. Consequently, promoting health-promoting lifestyles among occupational populations has become a critical public health priority.

Health-promoting lifestyle (HPL) refers to a pattern of proactive behaviors aimed at maintaining or enhancing health, Walker et al. described it as a multi-dimensional model that includes health awareness and related behaviors, covering health responsibility, physical activity, nutrition, interpersonal relations, spiritual growth, and stress management (4). Previous studies have identified various determinants of HPL (5–8), among which social support has been recognized as an important environmental factor. Social support refers to the emotional, informational, and instrumental assistance individuals receive from their social networks (9). Adequate social support can enhance self-efficacy, alleviate psychological stress, and provide necessary resources that facilitate the adoption and maintenance of healthy behaviors (10). Empirical evidence suggests that individuals with stronger social support are more likely to engage in positive health behaviors and maintain healthier lifestyles (11, 12). However, the mechanisms through which social support influences HPL remain insufficiently understood.

With the rapid development of digital technologies and the widespread use of online health information, digital health literacy (DHL) has emerged as a critical factor influencing individuals' health management. DHL refers to the ability to seek, understand, evaluate, and apply health information obtained from digital sources to make appropriate health decisions (13). Individuals with higher levels of DHL are more capable of accessing reliable health information, utilizing digital health services, and adopting evidence-based health behaviors. Previous studies have shown that digital health literacy is positively associated with various health outcomes and health-related behaviors (14–16).

Importantly, DHL may also be influenced by social environmental factors. From a socioecological perspective, health behaviors are shaped by the dynamic interaction between individual capabilities and environmental contexts (17). Social support may enhance DHL by providing informational resources, guidance in navigating digital environments, and encouragement to engage with digital health tools (18, 19). In turn, improved DHL may facilitate the translation of health information into actionable behaviors, thereby promoting healthier lifestyles.

Despite accumulating evidence on social support, DHL, and HPL, the underlying mechanisms linking these variables remain unclear. In particular, few studies have simultaneously examined the mediating role of DHL in the association between social support and HPL, especially within occupational populations characterized by unique work-related stressors and health risks.

Therefore, this study aims to examine the mediating role of DHL in the relationship between social support and HPL among occupational populations. By clarifying this mechanism, the study seeks to provide both theoretical insights and practical implications for developing integrated social and digital health interventions.

## 2 Materials and methods

### 2.1 Study population and data collection procedure

The study population comprised employed individuals aged 16–59 years in Huangpu District, Shanghai. Participants were excluded if they: (1) had severe physical functional impairments; (2) had diagnosed mental disorders; (3) were unwilling to participate in the study; or (4) were unable to independently complete or complete with assistance the questionnaire. Occupational categories were defined according to the *Occupational Classification Dictionary of the People's Republic of China (1999 edition)*. Sample size estimation was primarily based on the requirements for mediation analysis (20), assuming a small to medium effect sizes for both the a and b paths (a, b ≈ 0.14 ~ 0.39), approximately 150–700 participants are required to achieve a statistical power of 0.80 for detecting indirect effects using Bootstrap methods. To account for potential invalid responses and missing data, the final target sample size was conservatively set at approximately 840 participants.

A multistage cluster sampling method was employed. First, five subdistricts were randomly selected from Huangpu District, Shanghai. Second, workplaces including enterprises, public institutions, and commercial organizations were randomly selected within each subdistrict. Finally, all eligible employees in the selected workplaces were invited to participate until approximately 200 participants were recruited from each subdistrict.

This study was approved by the Ethics Committee of the Shanghai Huangpu District Center for Disease Control and Prevention (Approval No. 2024HPLL02). Written informed consent was obtained from all participants prior to data collection. Data were collected through on-site questionnaire administration conducted by trained investigators. To ensure data quality, attention-check items were included, and questionnaires with incorrect responses were excluded. In addition, questionnaires completed in less than 300 s were considered invalid, based on pilot testing results. A total of 1,058 questionnaires were distributed, of which 1,030 were valid, yielding an effective response rate of 97.35%.

### 2.2 Measurements

#### 2.2.1 Demographic characteristics

Demographic variables included sex, age, educational level, marital status, living arrangement, longest-held occupation and monthly household income per capita.

#### 2.2.2 Social support

Social support was assessed using the Social Support Rating Scale developed by Xiao Shuiyuan (21). The scale consists of 10 items covering three dimensions: objective support, subjective support, and support utilization. Total scores range from 0 to 66, with higher scores indicating higher levels of social support. Based on established criteria, scores < 33 indicate low support, 33–45 moderate support, and >45 high support. In this study, Cronbach's α was 0.87.

#### 2.2.3 Digital health literacy

DHL was measured using the Digital Health Literacy Scale developed by Zhao (22), based on instruments by van der Vaart and Drossaert (23) and Liu et al. (24). The scale includes four dimensions: digital health information acquisition (7 items), evaluation (5 items), application (5 items), and interaction (4 items), totaling 21 items. Each item is rated on a 5-point Likert scale (1 = strongly disagree to 5 = strongly agree). Higher scores indicate higher levels of DHL. In this study, the Cronbach's α coefficient for the overall DHL scale was 0.96, with subscale coefficients of 0.95 for digital health information acquisition, 0.92 for evaluation, 0.87 for application, and 0.84 for interaction.

#### 2.2.4 Health-promoting lifestyle

HPL was measured using the revised Health-Promoting Lifestyle Profile, adapted by Cao et al. (25) from the original profile developed by Walker et al. (4). The scale contains 40 items across six dimensions: health responsibility, nutrition, physical activity, stress management, interpersonal relations, and spiritual growth. Each item is rated on a 4-point Likert scale (1 = never to 4 = always). Total scores range from 40 to 160, with higher scores indicating a higher level of HPL. The HPL scale demonstrated excellent internal consistency in this study, with a Cronbach's α coefficient of 0.95 for the overall scale. The Cronbach's α coefficients for its subdimensions were 0.89 for health responsibility, 0.82 for nutrition, 0.89 for physical activity, 0.84 for stress management, 0.90 for interpersonal relations, and 0.93 for spiritual growth.

### 2.3 Statistical analysis

All analyses were conducted using SPSS version 27.0. Descriptive statistics were used to summarize participants' characteristics. Continuous variables are presented as mean ± standard deviation (SD), and group differences were examined using independent-samples *t*-tests or one-way ANOVA. Normality was assessed based on skewness and kurtosis values (absolute skewness < 2 and kurtosis < 7). Categorical variables are presented as frequencies and percentages and were compared using the chi-square test. Common method bias was assessed using Harman's single-factor test. Pearson correlation analysis was performed to examine associations among variables. The distributions of continuous variables were assessed using skewness and kurtosis values before statistical analyses. Variables were considered approximately normally distributed when the absolute values of skewness and kurtosis were within acceptable ranges. Multicollinearity among predictors was assessed using variance inflation factors (VIFs). The mediating effect of DHL was tested using Model 4 of the PROCESS macro. Bootstrapping with 5,000 resamples was applied to estimate standard errors and 95% confidence intervals (CI). A two-tailed *P* < 0.05 was considered statistically significant.

## 3 Results

### 3.1 Preliminary analysis

A total of 1,030 occupational participants were included in this study. Among them, 399 (38.74%) were male and 631 (61.26%) were female, with a mean age of 37.93 ± 10.29 years. The majority had a junior college or undergraduate education (73.69%) and were married or cohabiting (66.31%). The mean score of HPL was 100.21 ± 17.65. The mean scores of its dimensions were as follows: health responsibility (24.13 ± 5.36), nutrition (18.08 ± 3.53), physical activity (17.11 ± 4.86), interpersonal relations (13.86 ± 3.39), stress management (13.30 ± 3.12), and spiritual growth (13.74 ± 3.65).

Univariate analyses indicated that HPL scores differed significantly across several sociodemographic variables. Specifically, females reported higher HPL scores than males (*t* = −2.539, *P* = 0.019). Significant differences were also observed across age groups (*F* = 7.990, *P* < 0.001), with participants aged ≥46 years showing higher HPL levels compared with younger groups. Educational level was significantly associated with HPL (*F* = 14.383, *P* < 0.001), and individuals with a master's degree or above reported higher scores than those with lower educational attainment. Living arrangement also showed a significant effect (*F* = 5.797, *P* = 0.003), with participants living with family demonstrating higher HPL scores than those living with colleagues or friends. In addition, HPL varied significantly by occupation (*F* = 5.813, *P* < 0.001). Government/institution staff and clerical/office workers reported higher HPL scores compared with commerce/service personnel and general workers. Monthly household income per capita was also positively associated with HPL (*F* = 10.372, *P* < 0.001), with higher-income groups exhibiting better health-promoting lifestyles. No significant differences in HPL were observed across marital status (*P* = 0.154).

Regarding DHL, significant differences were observed by age (*F* = 2.921, *P* = 0.033) and monthly household income per capita (*F* = 5.347, *P* = 0.001). LSD *post hoc* comparisons indicated that participants aged 26–35 years had higher DHL scores than those aged ≥46 years (*P* = 0.004). In addition, participants with monthly household income per capita of 5,000–9,999 CNY, 10,000–14,999 CNY, and ≥15,000 CNY had higher DHL scores than those with income ≤ 4,999 CNY (all *P* < 0.01). No significant differences in DHL scores were found across gender, education level, marital status, living arrangement, or occupation.

Social support scores differed significantly across age (*F* = 13.748, *P* < 0.001), education (*F* = 3.874, *P* = 0.021), marital status (*F* = 99.606, *P* < 0.001), living arrangement (*F* = 63.235, *P* < 0.001), occupation (*F* = 10.332, *P* < 0.001), and monthly household income (*F* = 15.168, *P* < 0.001). *Post hoc* analyses showed that younger participants (< 25 years) had lower social support scores than participants aged 26–35, 36–45, and ≥46 years (all *P* < 0.05). Participants with a master's degree or above reported higher social support than those with secondary education or below (*P* = 0.006). Married/cohabiting participants had higher social support scores than unmarried and divorced/separated/widowed participants. Participants living with family reported higher social support scores than those living alone or with colleagues/friends. Significant differences were also observed across occupational groups, with government/institution staff reporting higher social support scores than several other occupational categories. Higher-income groups generally showed higher social support scores.

Detailed results are presented in Table 1, and *post hoc* comparisons for variables showing significant group differences are provided in Supplementary Table 1.

**Table 1:** Sociodemographic characteristics and differences in digital health literacy, social support, and health-promoting lifestyle scores among participants (*n* = 1,030).

Variables	*N* (%)	HPL		DHL		Social support	
		Mean ±SD	*t/F*	Mean ±SD	*t/F*	Mean ±SD	*t/F*
**Gender**			−2.539^**^		−1.919		−1.620
Male	399 (38.74)	98.46 ± 19.00		71.16 ± 16.62		37.81 ± 9.17	
Female	631 (61.26)	101.32 ± 16.66		73.14 ± 15.81		38.68 ± 7.93	
**Age (years)**			7.99^***^		2.921^*^		13.748^***^
≤ 25	118 (11.46)	95.45 ± 19.19		72.80 ± 19.09		35.18 ± 8.42	
26–35	339 (32.91)	98.42 ± 17.92		74.15 ± 16.13		37.09 ± 8.66	
36–45	343 (33.30)	101.08 ± 17.10		71.94 ± 15.36		39.65 ± 8.16	
≥46	230 (22.33)	103.99 ± 16.39		70.17 ± 15.48		39.87 ± 7.82	
**Education level**			14.383^***^		0.393		3.874^*^
Secondary or below	155 (15.05)	96.23 ± 18.50		71.49 ± 15.96		36.97 ± 8.37	
Junior college/undergraduate	759 (73.69)	99.90 ± 17.35		72.63 ± 16.13		38.40 ± 8.46	
Master's degree and above	116 (11.26)	107.53 ± 16.39		71.84 ± 16.59		39.83 ± 8.13	
**Marital status**			1.877		2.197		99.606^***^
Unmarried	315 (30.58)	98.74 ± 20.04		73.57 ± 17.61		33.39 ± 8.27	
Married/cohabiting	683 (66.31)	100.97 ± 16.61		72.02 ± 15.51		40.75 ± 7.48	
Divorced/separated/ widowed	32 (3.11)	98.47 ± 12.69		68.03 ± 13.75		35.81 ± 7.37	
**Living arrangement**			5.797^**^		0.493		63.235^***^
Living with family	740 (71.84)	101.22 ± 16.91		72.06 ± 16.09		40.01 ± 7.70	
Living alone	167 (16.21)	99.09 ± 18.26		73.08 ± 16.35		32.71 ± 8.83	
Living with colleagues/friends	123 (11.94)	95.61 ± 20.29		73.28 ± 16.33		36.00 ± 8.35	
**Longest-held occupation**			5.813^***^		0.710		10.332^***^
Government/institution staff	183 (17.77)	103.36 ± 18.10		73.61 ± 17.38		41.08 ± 8.53	
Commerce/service personnel	156 (15.15)	96.22 ± 16.26		71.28 ± 15.30		37.31 ± 9.20	
Professional/technical personnel	269 (26.12)	100.10 ± 17.73		72.23 ± 15.42		37.23 ± 7.79	
Clerical/office staff	333 (32.33)	101.72 ± 16.07		72.74 ± 15.79		39.03 ± 7.91	
General worker	89 (8.64)	95.42 ± 22.04		70.78 ± 18.45		35.33 ± 8.90	
**Monthly household income per capita (CNY)**			10.372^***^		5.347^***^		15.168^***^
≤ 4,999	193 (18.74)	95.33 ± 19.25		68.59 ± 17.10		35.13 ± 9.19	
5,000–9,999	430 (41.75)	99.68 ± 17.62		72.53 ± 16.24		38.36 ± 7.98	
10,000–14,999	210 (20.39)	101.33 ± 15.95		73.19 ± 15.80		39.20 ± 8.52	
≥15,000	197 (19.13)	104.96 ± 16.52		74.85 ± 14.78		40.55 ± 7.63	

Values are presented as mean ± standard deviation (SD). Independent-samples t-tests were used for comparisons between two groups, and one-way analysis of variance (ANOVA) for comparisons among three or more groups.

^*^P < 0.05; ^**^P < 0.01; ^***^P < 0.001.

### 3.2 Common method bias test

To examine the potential threat of common method bias, we conducted Harman's single-factor test by entering all measurement items into an unrotated exploratory factor analysis. The results showed that 13 factors had eigenvalues greater than 1, with the first factor accounting for 24.70% of the total variance. This value is well below the recommended threshold of 40% (26), indicating that common method bias was not a serious concern in this study.

### 3.3 Correlation analysis

The mean scores (± SD) were 23.99 ± 6.09 for acquisition, 17.42 ± 4.20 for evaluation, 17.83 ± 4.09 for application, and 13.13 ± 3.55 for interaction. The mean scores for DHL, social support, and HPL were 72.37 ± 16.15, 38.34 ± 8.44, and 100.21 ± 17.65, respectively.

Pearson correlation analysis indicated that DHL and its four dimensions, acquisition, evaluation, application, and interaction, were all significantly positively correlated with social support (*r* = 0.138–0.169, *P* < 0.001) and HPL (*r* = 0.250–0.315, *P* < 0.001). Social support was also positively correlated with HPL (*r* = 0.324, *P* < 0.001). Descriptive statistics and correlation coefficients are presented in Table 2.

**Table 2:** Descriptive analysis and correlations among variables (*n* = 1,030).

Variables	Mean ±SD	1	2	3	4	5	6	7
1. Acquisition	23.99 ± 6.09	—						
2. Evaluation	17.42 ± 4.20	0.835^***^	—					
3. Application	17.83 ± 4.09	0.775^***^	0.834^***^	—				
4. Interaction	13.13 ± 3.55	0.628^***^	0.640^***^	0.717^***^	—			
5. DHL	72.37 ± 16.15	0.928^***^	0.926^***^	0.919^***^	0.804^***^	—		
6. Social support	38.34 ± 8.44	0.164^***^	0.138^***^	0.169^***^	0.164^***^	0.176^***^	—	
7. HPL	100.21 ± 17.65	0.260^***^	0.279^***^	0.315^***^	0.250^***^	0.306^***^	0.324^***^	—

DHL, digital health literacy; HPL, health-promoting lifestyle. SD, standard deviation. Pearson correlation coefficients were calculated for bivariate correlations.

^***^P < 0.001.

### 3.4 Mediation analysis

**Figure 1:**
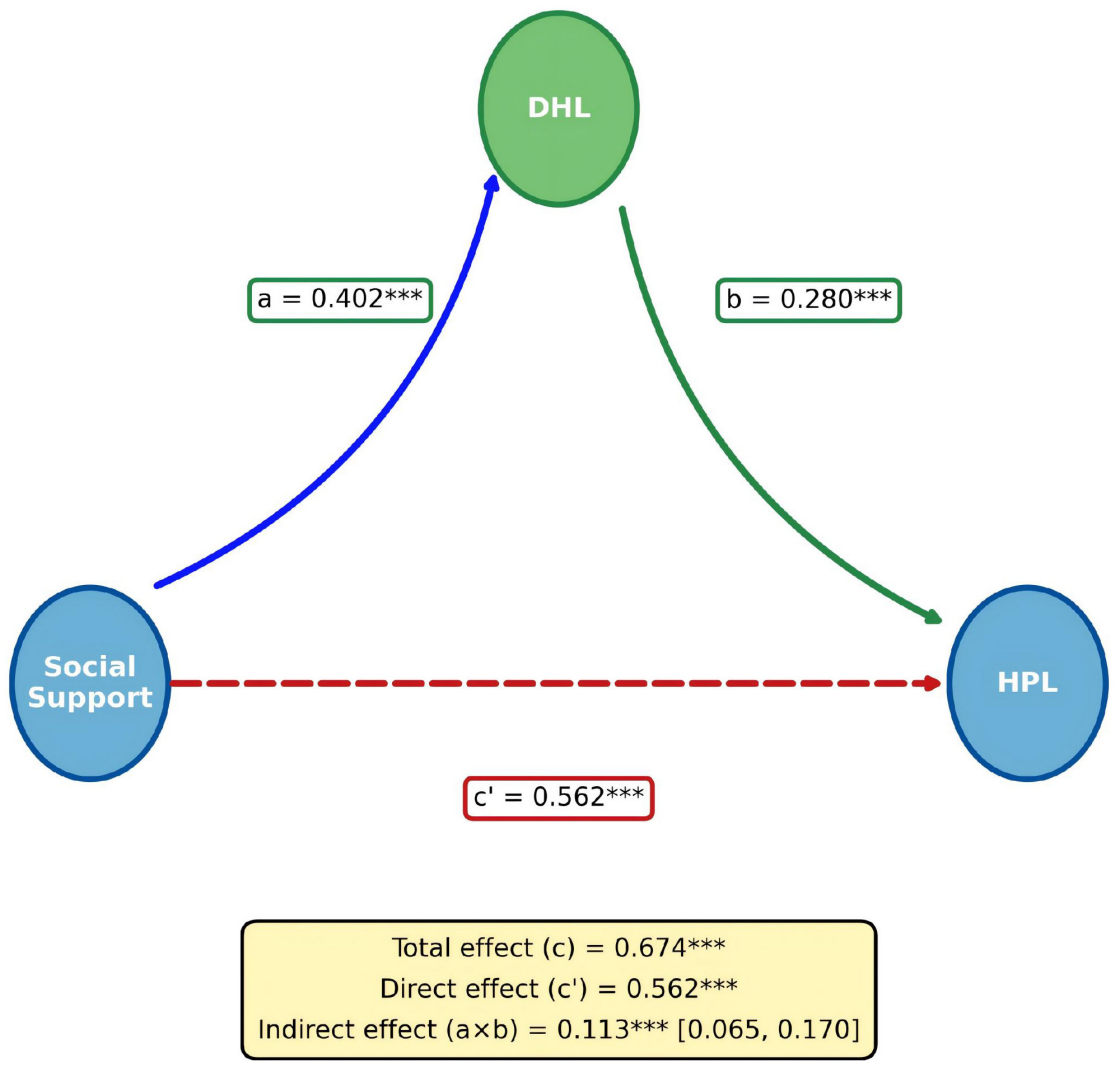
Mediation model of digital health literacy (DHL) in the association between social support and health-promoting lifestyle (HPL).

The mediating effect of DHL in the association between social support and HPL is presented in Table 3 and Figure 1. Social support was significantly positively associated with HPL in the total effect model (*B* = 0.674, *P* < 0.001, 95% CI [0.547, 0.802]). Social support was also significantly associated with DHL (*B* = 0.402, *P* < 0.001, 95% CI [0.279, 0.526]), and DHL was significantly positively associated with HPL (*B* = 0.280, *P* < 0.001, 95% CI [0.219, 0.341]). After including DHL in the model, the direct effect of social support on HPL remained significant (*B* = 0.562, *P* < 0.001, 95% CI [0.436, 0.687]). Bootstrap analysis indicated that the indirect effect of social support on HPL through DHL was significant (effect = 0.113, 95% CI [0.065, 0.170]).

**Table 3:** Mediating effect of digital health literacy (DHL) between social support and health-promoting lifestyle (HPL).

A. Direct and indirect paths						
Model and variable path	B	SE	t	95% CI	*R* ^2^	*F*
Total effect						
Social support → HPL	0.674	0.065	10.37^***^	[0.547, 0.802]	0.473	32.809
a path (X→mediator)						
Social support → DHL	0.402	0.063	6.40^***^	[0.279, 0.526]	0.257	9.020
b path (mediator→Y)						
DHL → HPL	0.280	0.031	8.98^***^	[0.219, 0.341]	0.404	24.877
B. Indirect effects (bootstrap results)						
**Path**	**Effect**		**Boot SE**		**Boot 95% CI**	
Social support → DHL → HPL	0.113		0.027		[0.065, 0.170]	

N = 1,030. All models controlled for demographics (sex, age, living arrangement, educational level, marital status, Monthly household income per capita, and Longest-held occupation). B = unstandardized coefficient. Bootstrap = 5,000 samples. Indirect effects are significant if 95% CI excludes zero. ^***^P < 0.001.

**Figure 2:**
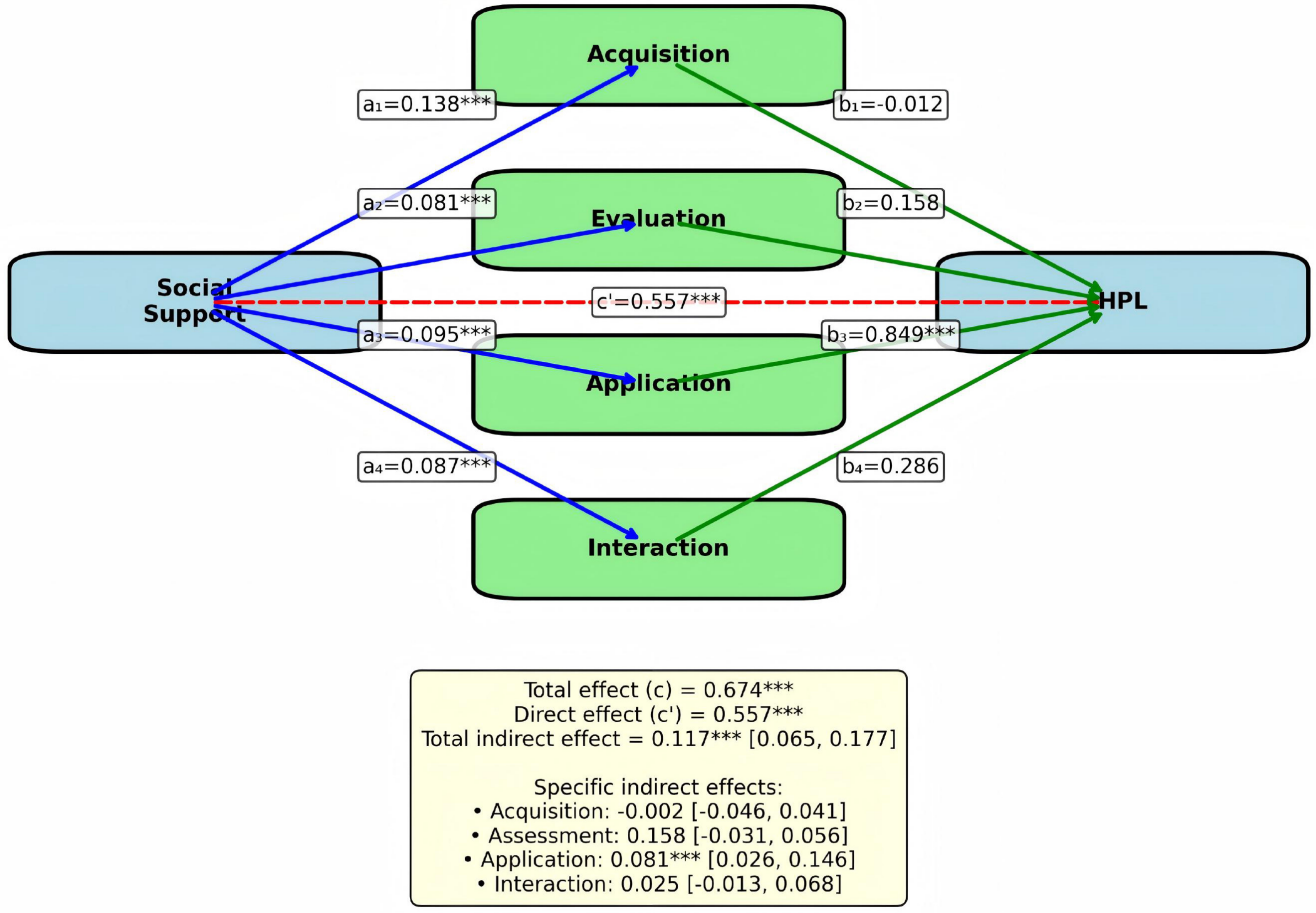
Parallel mediation model of digital health literacy dimensions in the association between social support and health-promoting lifestyle (HPL).

Parallel mediation analyses with the four dimensions of DHL are shown in Table 4 and Figure 2. The VIF values for the four DHL dimensions ranged from 2.121 to 4.655, suggesting that multicollinearity among predictors was not severe. Social support was significantly associated with all four dimensions, including acquisition (*B* = 0.138, *P* < 0.001), evaluation (*B* = 0.081, *P* < 0.001), application (*B* = 0.095, *P* < 0.001), and interaction (*B* = 0.087, *P* < 0.001). When the four mediators were simultaneously entered into the model, only application was significantly associated with HPL (*B* = 0.849, *P* < 0.001, 95% CI [0.365, 1.333]). The direct effect of social support on HPL remained significant (*B* = 0.557, *P* < 0.001, 95% CI [0.432, 0.683]). Bootstrap results further showed that the total iindirect effect was significant (effect = 0.117, 95% CI [0.065, 0.177]). Among the specific indirect effects, only the pathway through application was significant (effect = 0.081, 95% CI [0.026, 0.146]).

**Table 4:** Parallel mediation effects of digital health literacy (DHL) dimensions between social support and health-promoting lifestyle (HPL).

A. Direct and indirect paths						
Model and variable path	B	SE	*t*	95% CI	*R* ^2^	*F*
Total effects						
Social support → HPL (total effect)	0.674	0.065	10.37^***^	[0.547, 0.802]	0.404	24.877
a paths (X→M)						
Social support → acquisition	0.138	0.024	5.79^***^	[0.091, 0.185]	0.226	6.887
Social support → evaluation	0.081	0.016	4.95^***^	[0.049, 0.114]	0.230	7.128
Social support → application	0.095	0.016	6.02^***^	[0.064, 0.126]	0.274	10.332
Social support → interaction	0.087	0.014	6.30^***^	[0.060, 0.115]	0.239	7.737
b paths (M→Y)					0.483	25.413
Acquisition → HPL	−0.012	0.152	−0.08	[−0.310, 0.287]		
Evaluation → HPL	0.158	0.251	0.63	[−0.335, 0.650]		
Application → HPL	0.849	0.247	3.44^***^	[0.365, 1.333]		
Interaction → HPL	0.286	0.203	1.41	[−0.112, 0.685]		
Direct effect						
Social support → HPL	0.557	0.064	8.72^***^	[0.432, 0.683]		
B. Indirect effects (bootstrap results)						
Path	Effect		Boot SE		Boot 95% CI	
Total indirect	0.117		0.029		[0.065, 0.177]	
Social support → acquisition → HPL	−0.002		0.022		[−0.046, 0.041]	
Social support → assessment → HPL	0.013		0.022		[−0.031, 0.056]	
Social support → application → HPL	0.081		0.031		[0.026, 0.146]	
Social support → interaction → HPL	0.025		0.02		[−0.013, 0.068]	

N = 1,030. All models controlled for demographics (sex, age, living arrangement, educational level, marital status, Monthly household income per capita, and Longest-held occupation). B = unstandardized coefficient. Bootstrap = 5,000 samples. Indirect effects are significant if 95% CI excludes zero. ^***^P < 0.001.

## 4 Discussion

This study examined the relationships among social support, DHL, and HPL among occupational populations in Shanghai, and further explored the mediating role of DHL. The findings indicate that social support is positively associated with HPL, and that DHL partially mediates this relationship, providing evidence for the joint influence of social and digital factors on health behaviors in the digital era.

The overall level of HPL among the participants was moderate, which is generally consistent with findings reported among urban adult populations in China (27, 28). Among the six dimensions, health responsibility and nutrition scored relatively higher, whereas physical activity scored the lowest. This pattern may reflect increased awareness of disease prevention and diet, alongside limited opportunities for exercise due to sedentary occupational environments.

Significant differences in HPL were observed across sociodemographic characteristics. Females reported higher HPL scores than males, which is consistent with a study among employees in the Netherlands (29). This may be related to women's generally stronger health awareness and greater engagement in preventive health behaviors (30). Age was also positively associated with HPL, with older individuals demonstrating healthier lifestyles, possibly due to increased health concerns and accumulated health knowledge (24, 31). In addition, higher education and income levels were associated with better HPL, suggesting that socioeconomic advantages may facilitate access to health resources and support healthier behavior patterns (24, 32). Consistently, family functioning also exerted a positive influence on HPL in this study (33), highlighting the potential role of family support in reinforcing health-related behaviors (34). Occupational differences further suggest that work environment and job stability may influence opportunities for engaging in health-promoting activities (35).

Correlation analyses further supported the hypothesized relationships, showing that social support, DHL, and HPL were all positively associated. These findings are consistent with previous research indicating that social support plays an important role in promoting health behaviors by providing emotional, informational, and instrumental resources (36–39). Meanwhile, DHL was positively associated with HPL, suggesting that individuals with stronger abilities to access, evaluate, and use digital health information are more likely to adopt healthier lifestyles (40).

Importantly, mediation analysis revealed that DHL partially mediated the relationship between social support and HPL. This finding suggests that social support may promote healthier lifestyles not only directly, but also indirectly by enhancing individuals' digital health capabilities. Individuals embedded in supportive social environments may have more opportunities to access and exchange health information through both interpersonal communication and digital platforms, thereby improving their ability to translate information into health-related actions (41, 42).

Further analysis of DHL dimensions revealed that only the application dimension showed a significant mediating effect. Although social support was significantly associated with all four dimensions, acquisition, evaluation, and interaction did not significantly predict HPL when considered simultaneously. This finding highlights that the mere ability to obtain or understand health information may be insufficient for behavior change. Instead, the capacity to effectively apply health information in daily life appears to be an important pathway linking digital literacy to health behaviors. This result is consistent with previous research indicating that many digital health literacy interventions often focus primarily on information acquisition and comprehension, while neglecting the practical use of information (43). The application dimension reflects individuals' capacity to transform knowledge into actionable health practices, which may facilitate behavior change (44). However, given the moderate-to-high correlations among DHL dimensions and the potential influence of overlapping constructs, this finding should be interpreted cautiously and does not necessarily indicate that the application dimension is more important or superior to other DHL dimensions. Future studies are needed to further clarify the relative contributions of different DHL dimensions to health behaviors.

These findings have important implications for health promotion interventions. Interventions should move beyond improving access to health information and place greater emphasis on strengthening individuals' ability to apply such information in real-life contexts. Action-oriented strategies, such as goal setting, self-monitoring, and personalized feedback, may facilitate the integration of health knowledge into daily routines (45). In addition, digital tools such as mobile health applications and wearable devices may help bridge the gap between knowledge and action by providing real-time support and feedback (46).

Furthermore, enhancing social support within the workplace may amplify these effects by fostering environments that encourage information sharing, peer support, and collective engagement in health behaviors. Integrating social and digital approaches may therefore represent an effective strategy for promoting sustainable health behavior change among occupational populations.

Several limitations should be noted. First, the cross-sectional design precludes causal inference. Second, the study was conducted in a single urban district, which may limit generalizability. Third, self-reported data may be subject to information bias. Fourth, the exclusion of questionnaires completed within 300 s may have introduced selection bias. Although participants excluded by this criterion did not differ significantly from included participants in education level, occupation, or household income, differences were observed in age and several sociodemographic characteristics. Fifth, although a multistage cluster sampling approach was applied, the sample size calculation did not explicitly incorporate the design effect, which should be considered in future studies. Future studies should adopt longitudinal or experimental designs, include more diverse populations, and consider mixed-method approaches to further validate these findings.

## 5 Conclusion

This study demonstrates that social support positively predicts HPL among occupational populations, with DHL serving as a significant partial mediator. Notably, only the application dimension of DHL showed a significant mediating effect, highlighting the importance of translating health information into actionable behaviors. These findings suggest that workplace health promotion should strengthen both social support and individuals' ability to apply digital health information in daily life.

## Data Availability

The original contributions presented in the study are included in the article/Supplementary material, further inquiries can be directed to the corresponding author.
