# Supplementary material for: The mediating role of digital health literacy in the relationship between social support and health-promoting lifestyle among occupational populations: a cross-sectional study
Source: Front Public Health. 2026 Aug 31;14:1865814. doi: 10.3389/fpubh.2026.1865814 (PMC13572588; doi:10.3389/fpubh.2026.1865814)
Supplement: Supplementary file 1 [file Table_1.docx]

**Supplementary Table 1.** Differences in Health-Promoting Lifestyle, Digital Health Literacy and Social Support Scores Across Sociodemographic Characteristics

| **Variables** | **N (%)** | **HPL** | | | **DHL** | | | **Social support** | | |
| --- | --- | --- | --- | --- | --- | --- | --- | --- | --- | --- |
|  |  | **Mean ± SD** | **t/F** | **Post hoc (LSD)** | **Mean ± SD** | **t/F** | **Post hoc (LSD)** | **Mean ± SD** | **t/F** | **Post hoc (LSD)** |
| **Gender** |  |  | -2.539** | — |  | -1.919 |  |  | -1.620 |  |
| Male | 399 (38.74) | 98.46 ± 19.00 |  |  | 71.16 ± 16.62 |  |  | 37.81 ± 9.17 |  |  |
| Female | 631 (61.26) | 101.32 ± 16.66 |  |  | 73.14 ± 15.81 |  |  | 38.68 ± 7.93 |  |  |
| **Age (years)** |  |  | 7.99*** | a<c**, d***;  b<c*, d*** |  | 2.921* | b>d** |  | 13.748*** | a<b*, c***, d***; b<c***, <d*** |
| ≤25ᵃ | 118 (11.46) | 95.45 ± 19.19 |  |  | 72.80 ± 19.09 |  |  | 35.18 ± 8.42 |  |  |
| 26–35ᵇ | 339 (32.91) | 98.42 ± 17.92 |  |  | 74.15 ± 16.13 |  |  | 37.09 ± 8.66 |  |  |
| 36–45ᶜ | 343 (33.30) | 101.08 ± 17.10 |  |  | 71.94 ± 15.36 |  |  | 39.65 ± 8.16 |  |  |
| ≥46ᵈ | 230 (22.33) | 103.99 ± 16.39 |  |  | 70.17 ± 15.48 |  |  | 39.87 ± 7.82 |  |  |
| **Education level** |  |  | 14.383*** | a<b*, c***;  b<c*** |  | 0.393 |  |  | 3.874* | a<c** |
| Secondary or belowᵃ | 155 (15.05) | 96.23 ± 18.50 |  |  | 71.49 ± 15.96 |  |  | 36.97 ± 8.37 |  |  |
| Junior college/Undergraduateᵇ | 759 (73.69) | 99.90 ± 17.35 |  |  | 72.63 ± 16.13 |  |  | 38.40 ± 8.46 |  |  |
| Master’s degree and aboveᶜ | 116 (11.26) | 107.53 ± 16.39 |  |  | 71.84 ± 16.59 |  |  | 39.83 ± 8.13 |  |  |
| **Marital status** |  |  | 1.877 | — |  | 2.197 |  |  | 99.606*** | a<b***, b>c*** |
| Unmarried^a^ | 315 (30.58) | 98.74 ± 20.04 |  |  | 73.57 ± 17.61 |  |  | 33.39 ± 8.27 |  |  |
| Married/Cohabiting^b^ | 683 (66.31) | 100.97 ± 16.61 |  |  | 72.02 ± 15.51 |  |  | 40.75 ± 7.48 |  |  |
| Divorced/Separated/Widowed^c^ | 32 (3.11) | 98.47 ± 12.69 |  |  | 68.03 ± 13.75 |  |  | 35.81 ± 7.37 |  |  |
| **Living arrangement** |  |  | 5.797** | a>c*** |  | 0.493 |  |  | 63.235*** | a>b***, c***; b<c*** |
| Living with familyᵃ | 740 (71.84) | 101.22 ± 16.91 |  |  | 72.06 ± 16.09 |  |  | 40.01 ± 7.70 |  |  |
| Living aloneᵇ | 167 (16.21) | 99.09 ± 18.26 |  |  | 73.08 ± 16.35 |  |  | 32.71 ± 8.83 |  |  |
| Living with colleagues/friendsᶜ | 123 (11.94) | 95.61 ± 20.29 |  |  | 73.28 ± 16.33 |  |  | 36.00 ± 8.35 |  |  |
| **Longest-held occupation** |  |  | 5.813*** | a>b***, e***;  c>b*, e*;  d>b**, e** |  | 0.710 |  |  | 10.332*** | a>b***, c***, d**, e***; b<d*, c<d** d>e*** |
| Government/Institution staffᵃ | 183 (17.77) | 103.36 ± 18.10 |  |  | 73.61 ± 17.38 |  |  | 41.08 ± 8.53 |  |  |
| Commerce/Service personnelᵇ | 156 (15.15) | 96.22 ± 16.26 |  |  | 71.28 ± 15.30 |  |  | 37.31 ± 9.20 |  |  |
| Professional/Technical personnelᶜ | 269 (26.12) | 100.10 ± 17.73 |  |  | 72.23 ± 15.42 |  |  | 37.23 ± 7.79 |  |  |
| Clerical/Office staffᵈ | 333 (32.33) | 101.72 ± 16.07 |  |  | 72.74 ± 15.79 |  |  | 39.03 ± 7.91 |  |  |
| General workerᵉ | 89 (8.64) | 95.42 ± 22.04 |  |  | 70.78 ± 18.45 |  |  | 35.33 ± 8.90 |  |  |
| **Monthly household income per capita (CNY)** | | | 10.372*** | a<b**, c**, d***;  b<d***;  c<d* |  | 5.347*** | a<b**, c**, d*** |  | 15.168*** | a<b***, c***, d***; b<d** |
| ≤4,999ᵃ | 193 (18.74) | 95.33 ± 19.25 |  |  | 68.59 ± 17.10 |  |  | 35.13 ± 9.19 |  |  |
| 5,000–9,999ᵇ | 430 (41.75) | 99.68 ± 17.62 |  |  | 72.53 ± 16.24 |  |  | 38.36 ± 7.98 |  |  |
| 10,000–14,999ᶜ | 210 (20.39) | 101.33 ± 15.95 |  |  | 73.19 ± 15.80 |  |  | 39.20 ± 8.52 |  |  |
| ≥15,000ᵈ | 197 (19.13) | 104.96 ± 16.52 |  |  | 74.85 ± 14.78 |  |  | 40.55 ± 7.63 |  |  |

*Note.* Values are presented as mean ± standard deviation (SD). Independent-samples t tests were used for comparisons between two groups, and one-way analysis of variance (ANOVA) for comparisons among three or more groups.
